# Assessment of the oral health literacy and oral health behaviors among nurses in China: a cross-sectional study

**DOI:** 10.1186/s12903-022-02658-5

**Published:** 2022-12-13

**Authors:** Ran An, Wen-feng Chen, Siyu Li, Zitong Wu, Meizi Liu, Muhammad Sohaib

**Affiliations:** 1 Teaching and Research Section of Clinical Nursing, Xiangya Hospital of Central South University, Changsha, China; 2 Xiangya School of Nursing, Central South University, Changsha, China; 3 The First Affiliated Hospital of Zhengzhou University, Zhengzhou, China; 4 Children Hospital and Institute of Child Health, Multan, Pakistan; 5 Present Address: Xiangya Hospital of Central South University, Changsha, China

**Keywords:** Oral health literacy, Oral health behavior, Nurses, Oral health, Cross-sectional survey

## Abstract

**Purpose:**

Oral health is important for general health; nurses are expected to possess good oral health awareness and work together for public oral health promotion. The purpose of this study is to investigate oral health literacy (OHL)and oral health behaviors of nurses, and explore the association between oral health literacy with demographic variables and oral health behaviors.

**Methods:**

A cross-sectional study in a tertiary hospital was conducted using a short-form Health Literacy in Dentistry-14 (HeLD-14) and a 16-items oral health behaviors questionnaire. Information about the subjects’ demographic details including age, gender, place of residence, marital status, marital status, education level, monthly household income, working experience, etc. were collected. Independent sample t- test, One- way ANOVA, and multivariable regression were used to identify the association of oral health literacy with demographic variables and oral health behaviors.

**Results:**

A total number of 317 nursing nurses participated in the survey, with a mean OHL score of 36.72, SD10.531, 21.8% were categorized as good, 34.1% medium and 44.2% poor oral health literacy; monthly household income, self-rated oral health, brushing time, use of fluoride toothpaste, and regular oral examination were signficantly associated with OHL scores.

**Conclusion:**

The overall oral health literacy of the nurse population is at a moderate to low level. These findings may help to map and design an oral health education intervention to improve oral health literacy amongst nurses, especially nurses with low monthly household income and poor self-assessed oral health status. Nursing administrators and nursing educators should pay more attention to the oral health status of the nurse population.

## Introduction

Oral health is important for general health, numerous researches have shown the interaction between oral health, oral diseases and systemic complications like diabetes, digestive disease, stroke, cardiovascular disease, et al. [1]. Oral diseases are among the most prevalent diseases globally and have led to serious health and economic burdens [2]. Even though oral diseases are largely preventable, they persist with high prevalence, it was estimated that there were 3.5 billion cases globally [3].Poor oral health is a function of numerous factors including unhealthy diet, physical inactivity, tobacco and alcohol consumption, psychological stress, poor sanitation. WHO recommended the "Common Risk Factor Approach (CRFA)" for oral health promotion [4]. In addition to socio-economic factors, insufficient oral health care knowledge, limited access to dental services, and poor oral health care behavior are also barriers for oral health.

More recently, low oral health literacy (OHL) has emerged as a possible underlying mechanism for poor oral health [5]. According to the United States’ National Institute of Dental and Craniofacial Research, OHL is the degree to which people have the capacity to obtain, process, and understand basic oral and craniofacial health information and services needed to make appropriate health decisions [6]. It is generally accepted that oral health literacy is one of the important determinants of oral health [7].

Given the importance of oral health in the whole body and the high prevalence of oral diseases, the joint effort of clinicians and nurses is essential to people’s health, and it should be integrated as a part of the comprehensive health promotion [8]. Nurses are an important force in promoting the development of health undertakings, and as disseminators of health knowledge and advocates of healthy lifestyles and behaviors, their own OHL may have a significant impact on patients’ OHL, which affects patients' oral health status and cognition, attitudes ,and behaviors toward oral diseases [9]. Previous studies have highlighted the important associations between oral health literacy and oral health status [10, 11]. And there is some evidence that primary health care users with lower OHL levels have more severe periodontal disease [12].

Although there is a large body of research on oral health literacy in adults, adolescents, older adults [13, 14], pregnant women [15], and a few studies on medical students and nursing interns [16, 17], there is a paucity of research on the nurse population.

Elsewhere evidence shows that oral health literacy and oral health behaviors are influenced by socio-demographic characteristics [18]. Oral health literacy and oral health behaviors may vary with people's occupations, socioeconomic status, and overall health status. Self-rated oral health can provide a broader health perspective than clinically determined measures of dental status alone, according to a previous study [19]. Persons with higher oral health literacy tend to have better self-rated oral health and oral health-related quality of life [20, 21]. And self-reported oral health behaviors mainly included frequency of brushing teeth, brushing methods, usage of floss or mouth wash, frequency of visiting the dentist, the usage of use oral hygiene products, etc. [16, 22]. A self-assessed survey on the oral health behaviors of dental and medical students showed that the junior dental students showed highly significant improvement than their counterparts in the items about frequency of brushing teeth, brushing methods of vertical scrub or Bass technique, usage of floss or mouth wash [16]. Moreover, the study revealed that oral health and oral health behaviors exhibited gender differences, with men reporting poorer oral health, fewer dental visits, and poorer oral hygiene habits [23]. Socio-economic, individual oral health behaviors, and workforce characteristics are important considerations when assessing oral health outcomes [24]. However, the effect of socio-demographic characteristics on oral health literacy among nurses is not documented. Some prior studies have shown that the majority of nurses have poor knowledge regarding oral health across life span [8], and research found that nursing graduates have good basic oral health knowledge, while their knowledge of the oral-systemic disease connection and the value of an examination of the oral cavity were poor [25].

This study aims to investigate the oral health literacy level and oral health behaviors of nurses, determining the association between oral health literacy and socio-demographic characteristics, and to analyze differences in oral health behaviors of nurses with different oral health literacy.

## Methods

### Study design

A descriptive cross-sectional study design using a quantitative approach was distributed online through Questionnaire Star Platform. In this study, nurses' oral health behaviors and oral health literacy were examined. The self-assessed oral care health behaviors questionnaire included basic oral hygiene habits, knowledge of and use of oral hygiene care products, oral disease seeking behaviors, and  oral health knowledge awareness. The short-form Health Literacy in Dentistry questionnaire (HeLD-14) was used to assess oral health literacy.

### Study population

A convenience sample of participants was drawn from a tertiary hospital, The First People's Hospital of Chenzhou City, Hunan Province, China, from June to July 2022. Inclusion criteria: (1) Applicants must be involved in clinical nursing work and nursing management; (2) be at least 18 years old; (3) have worked for a minimum of a year. Exclusion criteria: (1) non-clinical nursing staff in the supply room, office, etc.; (2) nursing staff who were not on duty during the survey period due to sickness, maternity leave, etc. Respondents were informed of the survey and agreed to fill out the questionnaire voluntarily.

### Sampling

Since there is no survey on oral health literacy for nurses group, we refer to the survey on oral health literacy of adults [26], whose incidence of low level of oral health literacy is about 12.4%, and the survey on oral health literacy of university nursing students [17], whose incidence of low level of oral health literacy is about 12%, n = u^2^_α_*p* (1−*p*)/δ^2^, so this study takes *p* = 0.120 as the basis for estimating the sample size, n = the desired sample size when the population is greater than 10,000,δ is the allowable error, δ = 0.05 is set in this study, take α = 0.05, u = the standard normal deviate, usually set at 1.96 which corresponds to 95% confidence level, the final calculation of the sample content required for this study n ≈ 162, the effective response rate is calculated according to 80%, then the sample size required for this study is 202. There were 318 respondents in this survey, which met the minimum sample size requirement.

### Data collection procedure

After obtaining the approval of the the College of Nursing, Central South University, Nursing and Behavioral Medicine Research Ethics Review Committee (E20220116), as well as the study tools, data were collected from a tertiary hospital located in Chenzhou, Hunan Province, China, June 2022 and July 2022. The principal researcher visited the nursing directors of the Nursing Departments of the First People's Hospital of Chenzhou City to obtain permission for data collection. After we explained the purpose and methods of the study and obtained agreement from the Directors of Nursing, with the introduction of the head nurse, potential participants were approached by two investigators, informed of the purpose of the study and what it entailed, and told that their participation was voluntary and that they could terminate their participation at any time. If the nurses indicated interest, they were provided with an electronic version of the informed consent form and distribute a link to the questionnaire QR code, and to explain that the study was completed anonymously. The link to the electronic version of the questionnaire, with an introduction to informed consent before the questionnaire begins, can only be completed after clicking on the consent.

### The questionnaires

#### Oral health literacy scale

The short-form Health Literacy in Dentistry questionnaire (HeLD-14) was developed by Jones [27] based on the version of the Health Literacy in Dentistry questionnaire (HeLD-29). Chinese scholar Yan Wen [28] adapted and tested the Chinese version of the scale, we obtained the authorization of the authors. The Chinese version of the scale’s Cronbach’s alpha value was 0.89, and the test–retest reliability was 0.786, indicating an adequate level of inter-item reliability. The scale has 14 questions divided equally into seven dimensions. Each item was scored using a 5-point Likert scale ranging from 1 (‘without any difficulty’) to 5 (‘unable to do’). Analysis first converted the 5 points to 0, the 4 points to 1, the 3 points to 2, the 2 points to 3, the 1 point to 4, then the total score was calculated. The summary scores range from 0 to 56, with higher scores indicating higher oral health literacy.

#### Oral health behaviors questionnaire

After reviewing literature, group discussions, and consulting with experts, we developed the oral health behavior questionnaire based on the 5th edition of the World Health Organization (WHO) Basic Methods for Oral Health Surveys. A total of 16 items were included. It covers basic oral hygiene practices, knowledge and use of oral health care products, oral disease seeking behavior and oral health knowledge awareness. After a pre-survey of 30 people, the questionnaire was found to be simple and easy to understand, and it took 5–12 min to complete. The alpha value was 0.81, and the test–retest reliability was 0.86.

#### Socio-demographics questionnaire

The demographic information of participants including general demographic information such as age, gender, place of residence, marital status, education level, monthly household income, education level, job title, working experience, department, Self-rated oral health (SROH), etc.

### Statistical analysis

The data were analyzed using SPSS statistical software version24.0 (SPSS, Central south university, China). Data are presented as frequencies (counts and percentages) and mean ± standard deviation. The multiple stepwise regression analysis was used to analyze the factors and correlations of oral health literacy and oral health care behaviors. The level of significance was set at *p* < 0.05.

### Ethical approval and informed consent

All respondents gave informed consent prior to conducting the interview. A web-based informed consent form was presented to respondents before the formal questionnaire began. They read and ticked it to consent to participate. The study protocol was approved by the College of Nursing, Central South University, Nursing and Behavioral Medicine Research Ethics Review Committee (E20220116).

## Results

### Subjects

A total of 318 questionnaires were collected in this study, and after examination, 1 invalid questionnaire was excluded, and 317 valid questionnaires were collected. Among them, 13 were male and 304 were female, The average age of the participants was 32.25 years (SD = 7.10), most of them got bachelor's degree (81.4%) and married (71.3%), with more than 5 years of clinic experience (74.1%). Nearly one-third of them (32.8%) reported having a dental visit in the past year. More than half of the nurses rated their oral health status as average, while 13.6% rated their oral health status as poor, and 20.5% rated their oral health status as good. The demographic characteristics based on oral health literacy scores of the nurse participants are presented in Table 2.

### The oral health literacy scores

The oral health literacy scores of the 317 nurses ranged from 7 to 56 (36.72 ± 10.531) points. Among them, 140 (44.2%) of the nurses showed a low level (0–35 scores), 108 (34.1%) demonstrated a medium level (36–46 scores), and 69 (21.8%) achieved a high level of OHL score (above 46 scores). The two highest scoring dimensions were utilization and access to dental care, and the two lowest scoring dimensions were support and receptivity, as shown in Table 1.

**Table 1:** Oral health literacy scores of 317 nurses

Dimension	Score (mean ± SD)
Receptivity	4.72 ± 1.723
Understanding	5.45 ± 1.970
Support	4.66 ± 1.858
Economic barriers	4.83 ± 2.003
Access	5.69 ± 1.810
Communication	5.63 ± 1.831
Utilization	5.72 ± 1.776
Total oral health literacy score	36.72 ± 10.531

### Oral health literacy scores according to demographic characteristics

There were differences in oral health literacy scores according to demographic characteristics, among which there was no statistically significant (*p* > 0.05) comparison of scores by gender, education level, job title, and place of residence. Statistically significant scores (*p* < 0.05) were found for different age groups, work experience, department, marital status, monthly household income, whether they had seen a dentist in the last year, and self-rated oral health status, as shown in Table 2.

**Table 2:** Comparison of scores of oral health literacy among nurses with different demographic characteristics (n = 317)

Item	Number (%)	Total score (Mean ± SD)	*t*/*F* value	*P* value
Gender			− 0.816	0.415
Male	13 (4.1)	34.38 ± 11.836		
Female	304 (95.9)	36.82 ± 10.481		
Age group (years)			4.145	0.017
18 ~ 25	55 (17.4)	34.33 ± 9.355		
26 ~ 35	163 (51.4)	36.12 ± 10.990		
≥ 36	99 (31.2)	39.03 ± 10.024		
Education level			0.073	0.930
College and below	55 (17.4)	36.24 ± 11.129		
Bachelor's degree	258 (81.4)	36.81 ± 10.432		
Postgraduate and above	4 (1.3)	37.25 ± 11.087		
Title			1.197	0.311
Nurse	61 (19.2)	36.16 ± 9.689		
Nurse practitioner	91 (28.7)	36.15 ± 10.794		
Supervisor Nurse	134 (42.3)	36.58 ± 10.742		
Deputy chief nurse	31 (9.8)	40.06 ± 10.295		
Work experience (years)			4.041	0.018
1 ~ 5	82 (25.9)	35.29 ± 10.206		
6 ~ 10	97 (30.6)	35.23 ± 10.916		
≥ 11	138 (43.5)	38.62 ± 10.212		
Department			5.170	< 0.001
Internal medicine ward	92 (29.0)	35.46 ± 10.608		
Surgical ward	96 (30.3)	36.21 ± 9.998		
Emergency medicine	8 (2.5)	34.38 ± 10.253		
ICU	55 (17.4)	35.42 ± 10.709		
Dentistry	33 (10.4)	45.18 ± 8.461		
Other	33 (10.4)	36.00 ± 10.531		
Marital status			3.801	0.023
Married	226 (71.3)	37.68 ± 10.436		
Unmarried	88 (27.8)	34.52 ± 10.250		
Divorced or widowed	3 (0.9)	28.67 ± 16.862		
Place of residence			0.126	0.900
Urban	293 (92.4)	36.74 ± 10.593		
Rural	24 (7.6)	36.46 ± 9.943		
Monthly household economic income (RMB)			10.977	< 0.001
≤ 2000	9 (2.8)	34.44 ± 11.674		
2000–5000	85 (26.8)	33.39 ± 10.114		
5000–10,000	157 (49.5)	36.17 ± 9.694		
≥ 10,000	66 (20.8)	42.62 ± 10.644		
Whether to go to the dentist in the past year			3.746	0.005
Yes	104 (32.8)	39.83 ± 10.979		
No	213 (67.2)	35.20 ± 9.984		
Self-rated oral health			15.725	< 0.001
Poor	43 (13.6)	33.81 ± 10.032		
Fair	209 (65.9)	35.40 ± 10.113		
Good	65 (20.5)	42.88 ± 9.962		

A close relationship was found between nurses' oral health literacy scores and their age and years of work experience. Oral health literacy scores tended to increase with age and years of working experience, considering that as nurses' age and experience increased, their concern and understanding of health also grew. There were significant differences in oral health literacy scores among nurses in different departments, among which, nurses in dentistry scored much higher than other departments. Married nurses have the highest oral health literacy scores, followed by unmarried, and divorced or widowed nurses have the lowest scores.

The higher the monthly household income, the higher the oral health literacy score, which is consistent with the findings of Noor [18]. Those nurses who had seen a dentist in the last year had higher oral health literacy scores, and Self-rated oral health was closely associated with oral health literacy.

### Association among oral health behaviors and oral health literacy

Statistical analysis of nurses' oral health behaviors showed that 92.7% of nurses brushed twice or more times a day, 11.4% used horizontal brushing method, 54.3% used vertical brushing method, less used the rotary method (16.1%), 63.7% never or occasionally used dental floss, 68.2% never or occasionally used toothpicks, never or occasionally 46.2% rinsed their mouth after meals, while the percentage of nurses who did not use mouthwash was as high as 91.8%, only 44.8% used fluoride toothpaste, and 55.2% did not use or did not know about fluoride toothpaste. The percentages of regular oral examination and regular scaling were 51.7% and 38.5%, respectively. The frequency of replacing toothbrushes was only 63.1% for 2 ~ 3 months, 24.3% for once every six months, 42.6% for self-assessment of tooth decay, 75.4% for bleeding gums when brushing, 8.8% for bleeding teeth or swollen and painful gums, and 43.8% for no treatment. 65.6% of the reasons for going to the dentist were because of tooth pain. Among them, half of those with intolerable toothache and half of those with toothache are still tolerable, 14.6% of those who find tooth decay consult the dentist in time, only 8.8% of those who have regular checkup without discomfort, 10.7% of those who never take the initiative to learn about oral health, 71.3% of those who occasionally take the initiative to learn about oral health, and only 18.0% of those who often take the initiative to learn about oral health.

To analyze the association between oral health behaviors and OHL, the oral health behaviors were used as the independent variables and the oral health literacy scores as the dependent variable. There were no statistical differences in total OHL scores in terms of  the number of daily brushing, whether to use toothpicks, frequency of toothbrush replacement, and whether or not there were caries (*p* > 0.05). However, there was statistically significant difference between subjects with OHL scores in relation to  brushing method, brushing time, whether to use dental floss, rinsing after daily meals, using mouthwash, using fluoride toothpaste, regular oral examination, regular scaling, gum bleeding when brushing, treatment measures when brushing bleeding or gum swelling, reasons for visiting dentist, and whether to take the initiative to learn about oral health knowledge (*p* < 0.05). See Table 3.

**Table 3:** Associations between different oral health behaviors and oral health literacy

Item	Number (%)	Total score (Mean ± SD)	*t*/*F* value	*P* value
Daily brushing frequency (times)			1.831	0.162
1	23 (7.3)	34.96 ± 9.007		
2	266 (83.9)	36.52 ± 10.463		
≥ 3	28 (8.8)	40.11 ± 11.930		
Brushing method			4.178	0.006
Brush horizontally	36 (11.4)	35.42 ± 11.284		
Brush vertically	172 (54.3)	38.34 ± 10.352		
Brush in a circle	29 (9.1)	37.52 ± 10.789		
No fixed method	80 (25.2)	33.53 ± 9.842		
Brushing time (minutes)			9.899	< 0.001
≤ 1	59 (18.6)	33.22 ± 9.706		
1–2	156 (49.2)	35.78 ± 9.911		
> 2	102 (32.2)	40.18 ± 11.024		
Flossing			9.826	< 0.001
Never or occasionally	202 (63.7)	34.30 ± 10.473		
1 ~ 2 times per week	56 (17.7)	38.71 ± 8.470		
≥ 3 times per week	23 (7.3)	42.91 ± 10.483		
Once a day	20 (6.3)	44.70 ± 9.223		
More than 2 times a day	16 (5.0)	41.44 ± 8.548		
Use toothpicks			0.240	0.916
Never or occasionally	253 (79.8)	36.47 ± 10.651		
1–2 times per week	36 (11.4)	37.53 ± 11.355		
≥ 3 times per week	10 (3.2)	39.00 ± 7.318		
Once a day	14 (4.4)	36.86 ± 9.248		
More than 2 times a day	4 (1.3)	38.75 ± 9.215		
Rinse mouth after meals daily			8.487	< 0.001
Never or occasionally	160 (50.5)	34.41 ± 9.190		
1 time	45 (14.2)	35.42 ± 11.387		
2 times	60 (18.9)	39.83 ± 12.118		
More than 2 times	52 (16.4)	41.37 ± 9.505		
Use of mouthwash			− 3.817	< 0.001
No	291 (91.8)	36.06 ± 10.308		
Yes	26 (8.2)	44.12 ± 10.347		
Use of fluoride toothpaste			16.098	< 0.001
Yes	142 (44.8)	39.55 ± 11.038		
No	69 (21.8)	37.67 ± 9.297		
Don't know	106 (33.4)	32.31 ± 9.117		
Regular oral examination			7.252	< 0.001
No	164 (51.7)	32.88 ± 8.937		
Yes	153 (48.3)	40.84 ± 10.579		
Regular supragingival scaling			− 4.884	< 0.001
No	195 (61.5)	34.51 ± 9.627		
Yes	122 (38.5)	40.25 ± 10.983		
Frequency of toothbrush replacement			3.516	0.016
Never	27 (8.5)	35.11 ± 9.525		
half a year	77 (24.3)	3416 ± 9.158		
2 ~ 3 months	200 (63.1)	37.55 ± 11.025		
1 month	13 (4.1)	42.46 ± 9.033		
Whether there is tooth decay			2.789	0.063
Yes	135 (42.6)	36.86 ± 10.170		
No	145 (45.7)	37.54 ± 11.147		
Don't know	37 (11.7)	33.00 ± 8.625		
Bleeding gums when brushing teeth			2.873	0.036
Never	78 (24.6)	37.95 ± 11.941		
Sometimes (1 time/week)	191 (60.3)	37.23 ± 10.009		
Often (≥ 3 times/week)	34 (10.7)	32.71 ± 9.666		
Don't know or didn't care	14 (4.4)	32.71 ± 8.792		
When brushing bleeding or gums are swollen and painful			6.241	< 0.001
Don't need to treat	139 (43.8)	34.72 ± 9.993		
Go back to the dentist when you have time	125 (39.4)	38.10 ± 10.404		
Seek medical attention immediately	28 (8.8)	42.75 ± 12.668		
Take your own medicine or other	25 (7.9)	34.16 ± 7.983		
Reasons for going to the dentist			5.766	< 0.001
Never	35 (11.0)	34.34 ± 10.759		
Toothache is unbearable and medication does not work	104 (32.8)	34.92 ± 9.205		
Tooth pain is still tolerable	104 (32.8)	35.89 ± 10.353		
See a dentist when tooth decay is found	46 (14.6)	40.63 ± 9.595		
Regular checkup even without discomfort	28 (8.8)	43.00 ± 13.311		
Do you take the initiative to learn about oral health			30.252	< 0.001
Never	34 (10.7)	32.06 ± 9.205		
Occasionally	226 (71.3)	35.20 ± 9.758		
Often	57 (18.0)	45.51 ± 9.601		

*SD*, standard deviation

### Multivariate analysis of factors affecting nurses' oral health literacy

All variables which were found to be independently associated with OHL were included in the full multivariable model. The nurse’s OHL scores were used as the dependent variables, and the statistically significant entries in the univariate analysis were used as independent variables for multiple linear regression analysis. The ordered categorical variables (age group, work experience, monthly household income, self-rated oral health status, brushing time, dental flossing, rinsing after daily meals, whether to take the initiative to learn about oral health) were assigned values and substituted, the unordered multicategory variables (department, marital status, brushing method, fluoride toothpaste, gum bleeding when brushing, treatment measures when brushing bleeding or gum swelling, reasons for visiting dentist) were treated as dummy variables and substituted with original values for continuous variables, the dichotomous variables (dental visiting in the last year, usage of mouthwash, regular oral examination, regular supragingival scaling) are assigned a value of 1 for yes, and 0 for no. Among them, $2000 and below was used as reference level for monthly household income, self-rated poor oral health status was used as reference level for self-rated oral health status, brushing time less than one minute is the reference level for brushing time, the use of fluoride toothpaste was used as reference level for fluoride toothpaste, nurses with no regular oral examination as a reference level for regular oral examination. α in = 0.05, α out = 0.10, and the conditional likelihood ratio forward stepwise method was used. The results of linear regression indicated that there was a significant relationship between OHL between monthly household income, self-rated oral health, brushing time, use of fluoride toothpaste, and regular oral examination, as shown in Table 4. Nurses with higher monthly household income have higher OHL scores, and nurses who had better self-reported health status had higher OHL scores. Nurses who spent more time brushing their teeth each time had higher OHL scores, and nurses who had regular oral examination scored higher on OHL than those who did not have regular oral examination. All independent variable categories had covariate VIF values less than 5.

**Table 4:** Multifactor analysis of factors influencing oral health literacy among nurses

Independent variables	β	SE	β^′^	t	*p*
Constant	7.863	5.945		1.323	0.187
Monthly household income	2.554	0.729	0.184	3.503	0.001
Self-rated oral health status	2.043	0.961	0.113	2.125	0.034
Time to brush teeth each time	1.818	0.786	0.121	2.312	0.021
Don't know fluoride toothpaste	− 3.790	1.234	− 0.170	− 3.071	0.002
Regular oral examination	3.702	1.380	0.176	2.683	0.008

*R*^2^ = 0.366, adjusted *R*^2^ = 0.305, *F* = 5.948, *p* < 0.001

## Discussion

We investigated the oral health literacy, oral health behaviors, and self-assessed oral health status of nurses in a tertiary hospital in China. We found that the overall oral health literacy of the nurse population is at a moderate to low level.

The level of oral health literacy was reported to be higher in those with a higher level of monthly household income which has confirmed the results of other studies in other parts of the world [18, 20]. Families with higher monthly household income may be more likely to pay attention to their health since they have more financial means to pay for it, better living standard, and a stronger awareness of health. Therefore, it is suggested that more attention should be paid to the oral health status of low-income nurse populations.

Self-rated oral health is a comprehensive evaluation of the oral health of the study participants based on their health and overall subjective perceptions [20], overall, the better the self-rated oral health, the higher the oral health literacy scores, which is similar to the findings in some other populations, these studies suggest that self-rated oral health status is strongly associated with oral health behaviors [29], and is strongly associated with the incidence of periodontal disease [30], which indicated that the self-assessed oral health status can be used as a screening indicator to measure the oral health status of the general population, and can be combined with objective indicators through oral examinations for follow-up research.

The level of oral health literacy has a statistically significant relationship with oral health behaviors such as brushing time, use of fluoride toothpaste, and regular oral examination. Brushing is a basic technique to maintain oral health, and proper brushing can effectively remove most plaque, and research shows that the effect of brushing is closely related to the time spent brushing [31, 32]. Based on our data analysis, we found that brushing time was associated with oral health literacy, and it was found that nurses with higher oral health literacy brushed their teeth for a longer period of time. The percentage of those who brushed for 1 min or less each time was 18.6%, 49.2% brushed for 2 min, and 32.2% brushed for more than 2 min, which was much lower than the percentage of 83.1–96.7% of dental and medical students who brushed for for two minutes or more [16]. Oral health care professionals generally recommend at least 2 min brushing with an appropriate technique [33]. Some studies show that plaque removal increased with brushing time across the range studied, tending towards a maximum at longer brushing times, brushing for 180 s removed 55% more plaque than brushing for 30 s [34]. Besides, brushing time and brushing force have significant effects upon the level of plaque removal [35]. So, we should reinforce efforts to brush for longer periods of time, as increasing brushing time to the consensus minimum of 2 min so as to increase plaque removal to an extent likely to provide clinically significant oral health benefits.

An interesting finding in our study was the fact that many participants using toothpaste were unaware of the fluoride content of their toothpaste (33.4%), and nurses who did not know about fluoride toothpaste scored the lowest, the number of nurses who self-reported not using fluoride toothpaste accounted for 21.8%, which is less than satisfactory and should be addressed urgently. The use of fluoride toothpaste is one of the important ways to prevent caries [36]. The mechanism of fluoride toothpaste is mainly to reduce the solubility of tooth enamel and promote the remineralization of tooth enamel to inhibit the caries-causing bacteria, the latest evidence supports that fluoride toothpaste can reduce the caries rate and DMFT index (the Decayed, Missing and Filled Teeth) [37]. However, the results of the 4th National Oral Health Survey in China show that the caries rates of milk teeth of children aged 5 years is 70.9%, and the caries rate of permanent teeth of children aged 12 years is 34.5%, and the usage rate of fluoride toothpaste was 42.1% and 55% respectively. A study of Chinese secondary school students showed that the use of fluoride toothpaste was 7.5% [38]. It is important for people to be able to correctly determine the amount of fluoride they require based on the fluoride content of their water and dentifrices. Toothpastes with higher fluoride concentration increases the risk of fluorosis (enamel defects) in developing teeth. The lack of knowledge demonstrated by nurses about fluoride toothpaste may lead to worsening of fluorosis in endemic areas on the one hand, or to dental caries in areas with insufficient fluoride on the other.

Less than half of the nurses (48.3%) had regular oral examinations, and nurses who had regular oral examinations had significantly higher oral health literacy scores than those who did not have regular oral examinations. It was surprising to note that only 38.5% of nurses had regular supragingival scaling. Regular oral examinations and supragingival scaling are very important for maintaining oral health, but we regret to find that very few of the nursing community are able to do so. The finding that oral health behaviors were generally poor in China may be related to the large differences between domestic and foreign medical levels [39] and dental teaching contents of nursing professions, the late start of education and qualification of domestic dental nurses, and the great imbalance in the distribution of dental practitioners among the provinces [40]. In the future, we should strengthen the oral health education for nurses and the construction of the dental nursing team, establish a perfect education and training system, improve the oral health literacy level of nurses, and promote the physical and mental health and professional development of nurses.

## Limitations

This survey has some limitations. Firstly, our survey was conducted online instead of the offline questionnaire due to the COVID-19 Epidemic, so there may have been a respondent bias, as participants who completed the questionnaire may have been more concerned or interested in oral health issues. Secondly, cross-sectional studies take a snapshot of a specific condition at one point in time, which limits their ability to interpret causality. Further longitudinal studies or clinical trials may be required to extend the findings reported here. Furthermore, While the sample size met the power analysis, a convenience sample of nurses from one hospital in Hunan province was used, limiting the representativeness and generalizability of this study due to the vast size of China and the huge differences in economic and medical levels among different regions. Finally, the self-report questionnaire used to assess oral health behaviors may be overestimated or not reflect actual behaviors due to recall bias and social desirability. Therefore, the survey findings must be interpreted with caution.

## Conclusion

This study assessed the baseline oral health literacy of nurses in China. To our knowledge, there are no prior studies to assess nurses’ oral health literacy and oral health behaviors. This study provides baseline data on the level of oral health literacy of nurses. Baseline results showed low levels of general oral health literacy as measured by HeLD-14. As health knowledge disseminators and health managers of patients, nurses should improve their oral health literacy more in order to carry out effective output of oral health knowledge.

## Data Availability

All data generated or analyzed during the current study are available from the corresponding authors on reasonable request.
